# Protective Effects of Fucoxanthin on High Glucose- and 4-Hydroxynonenal (4-HNE)-Induced Injury in Human Retinal Pigment Epithelial Cells

**DOI:** 10.3390/antiox9121176

**Published:** 2020-11-25

**Authors:** Yi-Fen Chiang, Hsin-Yuan Chen, Yen-Jui Chang, Yin-Hwa Shih, Tzong-Ming Shieh, Kai-Lee Wang, Shih-Min Hsia

**Affiliations:** 1 School of Nutrition and Health Sciences, College of Nutrition, Taipei Medical University, Taipei 110301, Taiwan; da07108002@tmu.edu.tw (Y.-F.C.); d507104002@tmu.edu.tw (H.-Y.C.); 2 Department of Ophthalmology, Yang Ming Branch, Taipei City Hospital, Taipei 111024, Taiwan; DAC32@tpech.gov.tw; 3 Department of Healthcare Administration, Asia University, Taichung 41354, Taiwan; evashih@asia.edu.tw; 4 School of Dentistry, College of Dentistry, China Medical University, Taichung 404333, Taiwan; tmshieh@mail.cmu.edu.tw; 5 Department of Dental Hygiene, College of Health Care, China Medical University, Taichung 404333, Taiwan; 6 Department of Nursing, Ching Kuo Institute of Management and Health, Keelung City 203301, Taiwan; kellywang@tmu.edu.tw; 7 Graduate Institute of Metabolism and Obesity Sciences, College of Nutrition, Taipei Medical University, Taipei 110301, Taiwan; 8 School of Food and Safety, Taipei Medical University, Taipei 110301, Taiwan; 9 Nutrition Research Center, Taipei Medical University Hospital, Taipei 110301, Taiwan

**Keywords:** fucoxanthin, retinopathy, antioxidant

## Abstract

The incidence of diabetes mellitus is increasing due to the eating and living habits of modern people. As the disease progresses, the long-term effects of diabetes can cause microvascular disease, causing dysfunction in different parts of the body, which, in turn, leads to different complications, such as diabetic neuropathy, diabetic nephropathy, and diabetic retinopathy (DR). DR is the main cause of vision loss and blindness in diabetic patients. Persistent hyperglycemia may cause damage to the retina, induce the accumulation of inflammatory factors, and destroy the blood–retinal barrier function. Fucoxanthin (Fx) is a marine carotenoid extracted from seaweed. It accounts for more than 10% of the total carotenoids in nature. Fx is mainly found in brown algae and has strong antioxidant properties, due to its unique biologically active structure. This carotenoid also has the effects of reducing lipid peroxidation, reducing DNA damage, and preventing cardiovascular diseases as well as anti-inflammatory and anti-tumor effects. However, there is no relevant research on the protective effect of Fx in DR. Therefore, in this study, we explore the protective effect of Fx on the retina. Human retinal epithelial cells (ARPE-19) are used to investigate the protective effect of Fx on high glucose stress- (glucose 75 mM) and high lipid peroxidation stress (4-hydroxynonenal, 4-HNE (30 μM))-induced DR. The cell viability test shows that Fx recovered the cell damage, and Western blotting shows that Fx reduced the inflammation response and maintained the integrity of the blood–retinal barrier by reducing its apoptosis and cell adhesion factor protein expression. Using an antioxidant enzyme assay kit, we find that the protective effect of Fx may be related to the strong antioxidant properties of Fx, which increases catalase and reduces oxidative stress to produce a protective effect on the retina.

## 1. Introduction

The diabetes mellitus population has increased year after year and become a global issue [1]. With the progression of the disease, the long-term effect of diabetes causes microvascular disorders [2]. The disorder produces dysfunction in different sites of capillaries, such as retinal, renal, and neuronal [3]. This may turn to different complications: diabetic neuropathy, diabetic nephropathy, and diabetic retinopathy (DR) [3]. DR is a leading cause of vision loss and blindness in people with diabetes. Nearly forty percent of diabetes patients demonstrated different forms of DR [4], and diabetes is one of the top five reasons that the population older than 50 years has ophthalmic diseases [5]. The retinal pigment epithelium (RPE), located between the vascular choroids and the neurosensory retina, forms the protected outer blood–retinal barrier, which maintains the normal structural and function of retina. DR was found to primarily involve damage of the RPE [6].

Chronic hyperglycemia acts as the main pathology of DR, as the high glucose condition leads to the damage of the RPE and destroys the barrier function of the RPE [6]. The damage of cells causing the accumulation of reactive oxygen species (ROS) further enhances lipid peroxidation and generates 4-hydroxyalkenals, such as 4-HNE and 4-HDDE [7].

The damage of high glycaemia produces vascular endothelial dysfunction and the progression of atherosclerosis. Among then, the dysfunction will elevate the adhesion molecules, which play a critical role in microvascular complications. In diabetes, patients also demonstrated increased intercellular adhesion molecule-1 (ICAM-1) in the serum [8].

The hallmark of DR is the breakdown of the blood–retinal barrier (BRB). The BRB includes the retinal pigment epithelial and retinol endothelial cells, which maintain the structure of cell-to-cell interactions [9]. A DR in vitro model showed that the high glucose condition decreased the ZO-1 level and increased the barrier permeability [10]. This produced the chronic pathology of DR and diabetic macular edema (DME).

Fucoxanthin (Fx) is a carotenoid that is mainly found in brown seaweeds. The unique bioactivities of fucoxanthin are related to the unique structural characteristics. Fx has high antioxidative activity, greater than α-tocopherol [11]. In addition to being an antioxidant [12], Fx was found to lower the lipid peroxidation [13] and possess anti-fungal [14,15], anti-inflammatory [16], anti-hyperuricemia [17], and anti-tumor [18] effects. Fx was further researched regarding DNA damage and in having a protective effect on cardiovascular diseases [19]. However, there is no study on the protection of fucoxanthin in glycaemia-related retinopathy. In this study, we investigated the mechanism of fucoxanthin on the protection in RPE.

## 2. Materials and Methods

### 2.1. Reagents

High-stability fucoxanthin (HS Fucoxanthin, HSFUCO, Fx) was obtained from Hi-Q Marine Biotech International Ltd. (Taipei, Taiwan), Glucose was obtained from Sigma-Aldrich (St. Louis, MO, USA). 4-HNE was obtained from Cayman (Ann Arbor, MI, USA).

### 2.2. Human RPE Cell Culture

The human arising retinal pigment epithelia (RPE) ARPE-19 cell line was purchased from the American Type Culture Collection (ATCC, Manassas, VA, USA). ARPE-19 cells were maintained in Dulbecco’s Modified Eagle’s Medium (DMEM)/F12 medium (CASSION, Taichung City, Taiwan) supplemented with 10% fetal bovine serum (FBS; CORNING, Manassas, VA, USA), 100 units/mL of penicillin, 100 μg/mL of streptomycin (CORNING, Manassas, VA, USA), sodium bicarbonate (2.438 g/L; BioShop, Burlington, ON, Canada), and 4-(2-hydroxyethyl) piperazine-1-ethanesulfonic acid (HEPES; 5.986 g/L; BioShop) in a humidified incubator (37 °C at 5% CO_2_).

### 2.3. 3-(4,5-Dimethylthiazol-2-yl)-2,5-Diphenyltetrazolium Bromide (MTT) Assay

The cell viability was developed using an MTT assay (Abcam, MA, USA). ARPE19 cells were seeded in 96-well plates (3000 cells/well) with serum free starvation for 18 h and then treated with 4-HNE or high glucose combined with fucoxanthin (mg/mL) for 24 or 72 h. After the treatment, serum-free cultured medium with 1 mg/mL of MTT was incubated for an additional 3 h; subsequently, the media was removed. Crystal formazan was dissolved in 100 μL/well dimethyl sulfoxides (DMSO; ECHO Chemical Co. Ltd., Taipei, Taiwan). The optical density was measured by using a VERSA Max microplate reader (Molecular Devices, San Jose, CA, USA) at 570 and 630 nm.

### 2.4. Cell Counting and Propidium Iodide (PI) Staining

ARPE-19 cells were seeded in a 96-well or 12-well plate. After attaching overnight, the cells were treated with serum free starvation for 16 h then treated with 4-HNE or high glucose combined with fucoxanthin (mg/mL) for 24 or 72 H. The cells were detected using trypsin. Cell suspensions were mixed with 0.4% trypan blue solution (Gibco, Grand Island, NY, USA). The cells were counted using a hemocytometer under an inverted phase-contrast microscope at 200× magnification. Propidium iodide (PI) stock solution (500 μg/mL, Sigma-Aldrich) was prepared and dissolved in sterile water. The PI (1 μg/mL) solution was used to stain the samples for 1 h to measure the DNA damage, and inverted phase-contrast microscopy was used for fluorescence capture at 200× magnification.

### 2.5. Protein Extraction and Western Blot

Cells were lysed in radioimmunoprecipitation assay (RIPA) lysis buffer with protease and phosphatase inhibitors (Roche, Mannheim, Baden-Württemberg, Germany). The protein was quantitated using a bicinchoninic acid (BCA) assay and using sodium dodecyl sulfate polyacrylamide gel electrophoresis (SDS-PAGE) with 85 voltage and 2 h transfer to a polyvinylidene fluoride (PVDF) membrane. We used 5% bovine serum albumin (BSA) solution to block for 1 h. The membranes were incubated with primary antibodies—Poly (ADP-ribose) polymerase (PARP) (1:1000; Cell Signaling, Boston, MA, USA), Glyceraldehyde 3-phosphate dehydrogenase (GAPDH) (1: 10,000; Proteintech, Rosemont, IL, USA), Intercellular Adhesion Molecule 1 (ICAM-1) (1:1000, SAB, MD, USA), Zonula occludens-1 (ZO-1) (1:1000, Affinity, Cincinnati, OH, USA), occludin (1:1000, Affinity), nuclear factor erythroid 2 (Nrf2) (1:1000, Genetex, CA, USA), BCL2 Associated X (Bax) (1:1000, cell signaling), and B-cell lymphoma 2 (Bcl-2) (1:500, santacruz, Santa Cruz, CA, USA)—at 4 °C overnight and cultured with a horseradish peroxidase (HRP)-conjugated secondary antibody (1:5000–10,000) for 2 h. The signal was captured using an image analysis system (UVP BioChemi, Analytik Jena US, Upland, CA, USA). The band densities were determined as arbitrary absorption units using the Image-J software program version 1.52 t (NIH, Bethesda, MD, USA). The expression level of these target proteins was analyzed in three individual experiments. In the same quantitative protein sample, two different molecular weights of the target protein were determined in the same gel. The membrane was cut into two fractions and incubated with two different antibodies at the same time.

### 2.6. Immunofluorescence

After the treatments, the cells were fixed by 4% paraformaldehyde for 10 min at room temperature, permeabilized with 0.5% Triton X-100 in PBS for 10 min, and then blocked with 5% bovine serum albumin (BSA) in TBST for 30 min at room temperature. ARPE19 cells were incubated with anti-ZO1 (1:200, affinity) diluted in 5% BSA overnight at 4 °C, followed by Alexa Fluor 546-goat anti-rabbitImmunoglobulin G antibodies for 1 H at room temperature. The cells were visualized under a fluorescence microscope.

### 2.7. 2,2-Diphenyl-1-Picrylhydrazyl (DPPH) Assay

We used a commercial kit (Dojindo, Japan) and followed the instructions from the manufacturer and a previous study [20]. The scavenging activity was detected using 100 μL 2,2-diphenyl-1-picrylhydrazyl (DPPH) solution mixed with the sample in a 96-well microplate and incubated at room temperature for 30 min. The absorbance was measured at 517 nm using a VERSA Max microplate reader (Molecular Devices, San Jose, CA, USA) and using the following formula:

Scavenging activity (%) = [control − sample/control] × 100.(1)

### 2.8. Antioxidant Ability Measurement

After the treatments, we used a commercial kit (Catalase assay kit, Cayman, UK, 707002) and followed the manufacturer’s description to analyze the catalase change. Briefly, the cells were cultured in a 6-cm dish, and then the treatment cells were harvested, and the homogenies were followed using the manufacturer’s instructions. We read the absorption at 540 nm using a VERSA Max microplate reader. To analyze the reactive oxygen species density, the cells were cultured in 96-well plates (3000 cells/well). After the treatments, we incubated the samples with 25 μM 2′,7′-dichlorofluorescin diacetate (DCFDA, Cayman) for 30 min. A fluorescence microscope was used to capture the fluorescence. We used image J to quantify the ROS density in single cells. For each group, we randomly chose three different fields and three single cells to calculate the ROS density.

### 2.9. Statistical Analysis

The data are expressed as the mean ± standard deviation SD). Student’s t-test was used for comparisons between two groups. One-way ANOVA tests were performed to compare multiple groups followed by Tukey’s post hoc test. A *p*-value of 0.05 or lower was considered significant in all experiments. All analyses were performed using GraphPad Prism 8.0. The p value is presented as *, *p* < 0.05; **, *p* < 0.01; ***, *p* < 0.001; or #, *p* < 0.05; ##, *p* < 0.01; and ###, *p* < 0.001.

## 3. Results

### 3.1. Fucoxanthin Prevents 4-HNE- and High Glucose-Mediated Suppression of ARPE-19 Cell Viability

**Figure 1:**
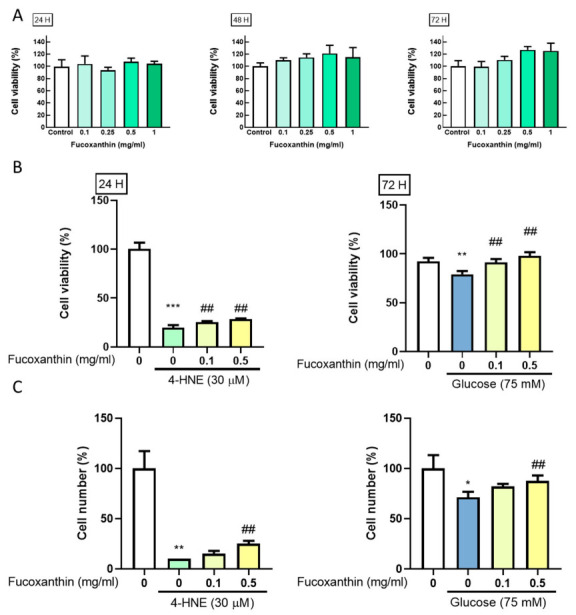
The effects of high glucose, fucoxanthin, and the combination on ARPE19 cell viability. ARPE19 cells were cultured in a 96-well plate (3000 cell/well) after starvation with serum free Dulbecco’s Modified Eagle’s Medium (DMEM) F12 for 18 h. The cells were treated with different concentrations of (**A**) fucoxanthin for 24, 48, or 72 h; combined with fucoxanthin induced by 30 µM 4-hydroxynonenal (4-HNE) for 24 h or 75 mM glucose for 72 H (*n* = 4). Used (**B**) MTT assay and (**C**) cell counting for analysis of the proliferation change. *, *p* < 0.05; **, *p* < 0.01; and ***, *p* < 0.001 compared with the non-treated group. ##, *p* < 0.01 compared with the induced group (glucose 75 mM/4-HNE 30 µM).

We simulated the pathology of diabetes retinopathy using high lipid peroxidation and high glucose condition. We used an MTT assay to evaluate the cell viability change and simulate the damage in RPE cells. ARPE-19 cells were seeded in a 96-well plate for 24 h. After starvation for 18 h, fucoxanthin treatment was added for 24, 48, and 72 h. The results showed that fucoxanthin did not affect the cell viability of ARPE-19 (Figure 1A). After finding the effective concentration, fucoxanthin pretreated was added for 5 h and combined with 4-HNE or high glucose for 24 or 72 h. We found that fucoxanthin had a protective effect on 4-HNE- or high-glucose-mediated suppression cell viability (Figure 1B) and had a consistent pattern in cell number (Figure 1C). This indicates that fucoxanthin can alleviate 4-HNE- and high glucose-induced cell proliferation changes.

### 3.2. Fucoxanthin Prevented 4-HNE- and High Glucose-Mediated ARPE-19 Cell Morphology Changes and DNA Damage

**Figure 2:**
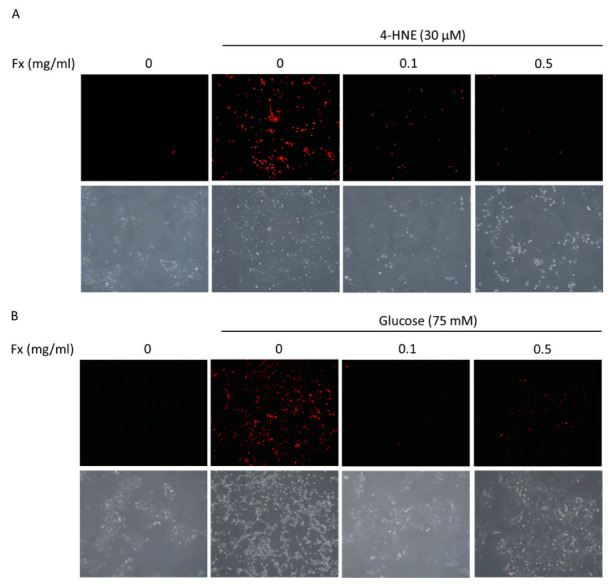
Effects of fucoxanthin (Fx) on 4-HNE- and high glucose-induced ARPE19 cell propidium iodide (PI) staining and morphology changes. ARPE19 cells were cultured in DMEM F12 for 24 h after serum free starvation for 18 h. This was combined with fucoxanthin induced by (**A**) 30 µM 4-hydroxynonenal (4-HNE) for 24 h or (**B**) 75 mM glucose for 72 h. We used PI staining and photographed with a microscope at 20× magnification.

To further confirm the fucoxanthin protective effect in ARPE-19, we examined the morphology changes and used PI-staining to examine the DNA damage after the treatment. The results showed that the treatment of 4-HNE (Figure 2A) and high glucose (Figure 2B) caused DNA damage and morphology changes in ARPE-19 cells. Fucoxanthin was shown to reverse the damage and recover the morphology of ARPE-19 cells.

### 3.3. Fucoxanthin Decreased the Apoptosis-Related Protein Expression

**Figure 3:**
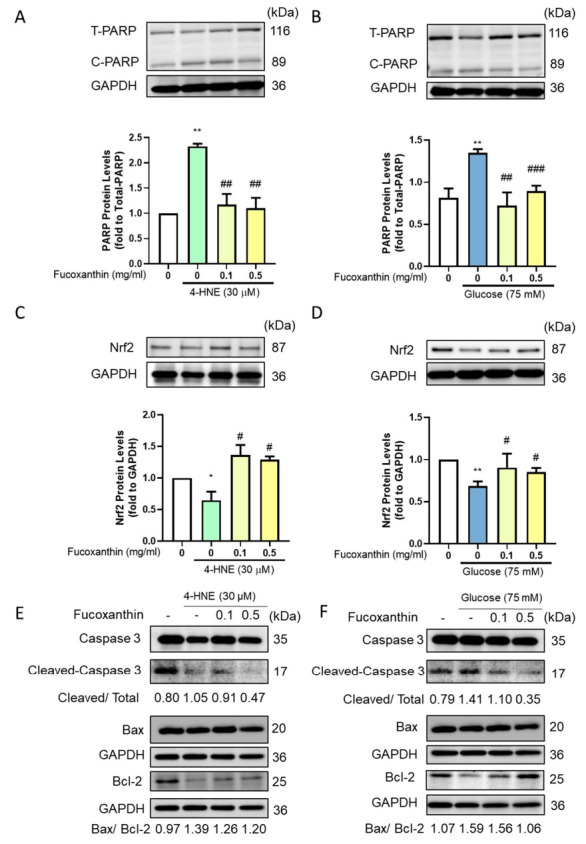
Effects of fucoxanthin on 4-HNE- and high glucose-induced ARPE19 apoptosis-related protein expression. We used Western blotting to analyze the (**A**,**B**) PARP; (**C**,**D**) Nrf2 protein; and (**E**,**F**) apoptosis-related protein expression (*n* = 4). *, *p* < 0.05; and **, *p* < 0.01 compared with the non-treated group. #, *p* < 0.05; ##, *p* < 0.01; and ###, *p* < 0.001 compared with the induced group (glucose 75 mM/4-HNE 30 µM). T-PARP, total PARP; C-PARP, cleaved PARP.

With the significant cell damage and the decrease of cell viability, we further evaluated the apoptosis-related protein expression using a Western blot. The induced group showed a significant increase in cleaved form PARP protein expression, and fucoxanthin treatment significantly reversed the cleaved form PARP protein expression (Figure 3A,B). Since the pathology of diabetes retinopathy is the increase of oxidative stress, we further examined Nrf-2, which is related with apoptosis progression and the antioxidant enzyme ability. The results showed that the induced group had significantly lower Nrf2 protein expression and that the fucoxanthin treatment significantly increased the protein expression (Figure 3C,D). The caspase 3 cleaved form increased after 4-HNE and high glucose, while fucoxanthin recovered the cleaved form of caspase 3 and decreased the Bax/Bcl-2 ratio to reduce the apoptosis progression (Figure 3E,F), providing the recovery of the apoptosis and antioxidant ability.

### 3.4. Effect of Fucoxanthin on the Protection of the Endothelial Function and Completeness in the Blood–Retinal Barrier (BRB)

**Figure 4:**
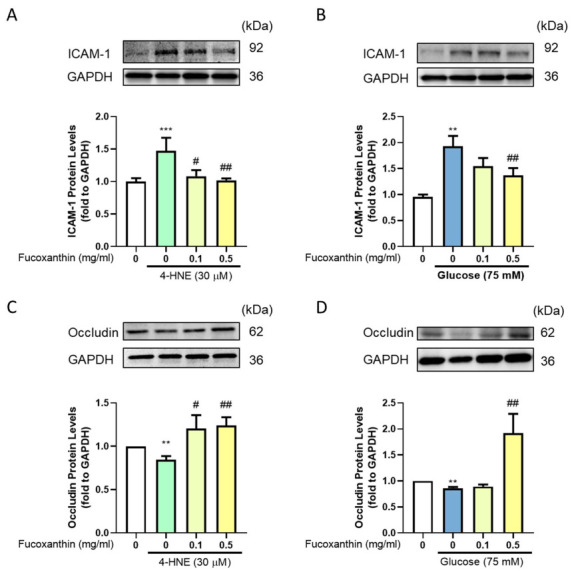
Effects of fucoxanthin on 4-HNE- and high glucose-induced ARPE19 blood–retinal barrier protein expression. ARPE19 cells were cultured in DMEM F12 for 24 h after serum free starvation for 18 h. This was combined with fucoxanthin induced by 30 µM 4-hydroxynonenal (4-HNE) for 24 h or 75 mM glucose for 72 h. We used Western blotting to analyze the (**A**,**B**) ICAM-1 and (**C**,**D**) occludin protein expression (*n* = 3). **, *p* < 0.01; and ***, *p* < 0.001 compared with the non-treated group. #, *p* < 0.05; and ##, *p* < 0.01 compared with the induced group (glucose 75 mM/4-HNE 30 µM).

The blood–retinal barrier forms a complete structure in ARPE-19 cells. In the barrier structure, the tight junction plays an important role. To examine the completeness of the structure, we evaluated the adhesion molecules and tight junction-related protein expression. We used Western blotting to evaluate the protein expression. The results showed that fucoxanthin successfully recovered the 4-HNE-, and high glucose-induced adhesion molecules increased by decreased ICAM-1 protein expression (Figure 4A,B) and increased the occludin protein expression (Figure 4C,D) after fucoxanthin treatment.

### 3.5. The Protective Effect of Fucoxanthin on Tight Junction Connections

**Figure 5:**
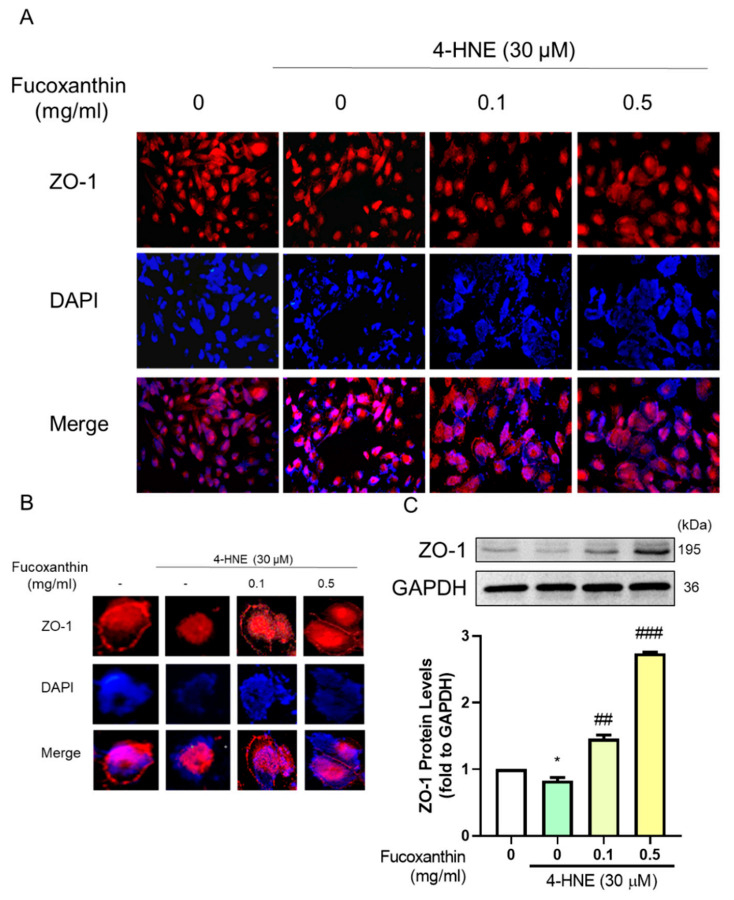
Effects of fucoxanthin on 4-HNE-induced ARPE19 tight junction-related protein expression. ARPE19 cells were cultured in DMEM F12 for 24 h after serum free starvation for 18 h. This was combined with fucoxanthin induced by 30 µM 4-hydroxynonenal (4-HNE) for 24 h. We used (**A**) immunofluorescences, (**B**) the single cell structure completeness change, and (**C**) Western blotting to analyze the ZO-1 protein expression (*n* = 4). *, *p* < 0.05 compared with the non-treated group. ##, *p* < 0.01; and ###, *p* < 0.001 compared with the induced group (4-HNE 30 µM). We used a microscope at 40× magnification.

**Figure 6:**
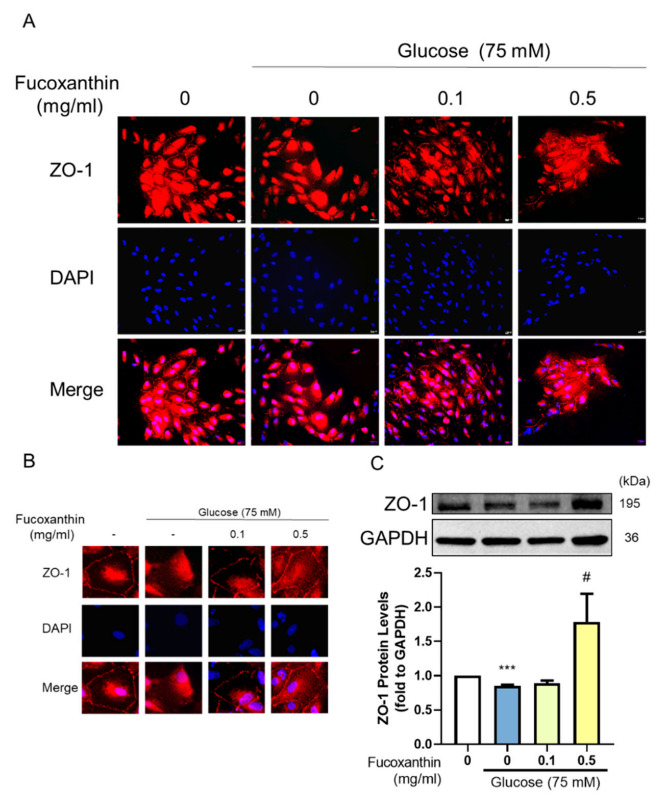
Effects of fucoxanthin on high glucose-induced ARPE19 tight junction-related protein expression. ARPE19 cells were cultured in DMEM F12 for 24 h after serum free starvation for 18 h. This was combined with fucoxanthin induced by 75 mM glucose for 72 h. We used (**A**) immunofluorescences, (**B**) the single cell structure completeness change, and (**C**) Western blotting to analyze the ZO-1 protein expression (*n* = 4). ***, *p* < 0.001 compared with the non-treated group. #, *p* < 0.05 compared with the induced group (glucose 75 mM). We used a microscope at 40× magnification.

The barrier completeness is based on tight junction connections. We used Western blotting and immunofluorescence to measure the completeness of the barrier. The results showed that fucoxanthin alleviated the damage given by the 4-HNE. We used immunofluorescence to show the completeness of the structure on the cell membrane by estimating the ZO-1 expression (Figure 5A,B), which was consistent with the measured ZO-1 protein expression (Figure 5C). Fucoxanthin also showed a protective effect on high glucose-induced tight junction breakdown with the structure completeness (Figure 6A,B) and protein expression of ZO-1 (Figure 6C). Fucoxanthin significantly increased the ZO-1 expression and recovered the structure, demonstrating the recovery ability of fucoxanthin in diabetes retinopathy.

### 3.6. The Antioxidant Ability of Fucoxanthin in 4-HNE- or High Glucose-Induced Diabetes Retinopathy

**Figure 7:**
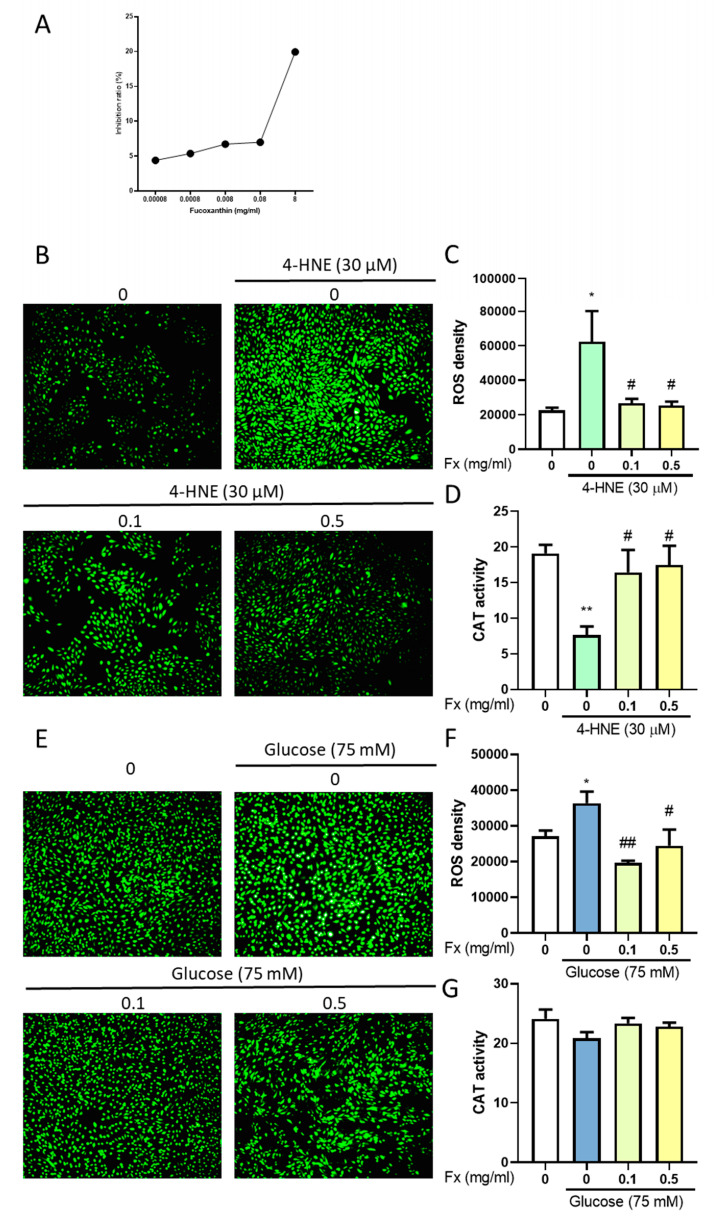
Effects of fucoxanthin (Fx) on 4-HNE- and high glucose-induced ARPE19 antioxidant ability change. (**A**) We used a 2,2-diphenyl-1-picrylhydrazyl (DPPH) assay to evaluate the antioxidant ability of fucoxanthin. Treated with 4-HNE to measure the DCFHA fluorescence (**B**) image, (**C**) level, and (**D**) antioxidant enzyme activity or high glucose-induced oxidative DCFHA fluorescence (**E**) image, (**F**) level, and (**G**) antioxidant enzyme activity change. ARPE19 cells were cultured in DMEM F12 for 24 h after serum free starvation for 18 h. This was combined with fucoxanthin induced by 30 µM 4-hydroxynonenal (4-HNE) for 24 h or 75 mM glucose for 72 h (*n* = 3–5). *, *p* < 0.05; and **, *p* < 0.01 compared with the non-treated group. #, *p* < 0.05; and ##, *p* < 0.01 compared with the induced group (4-HNE 30 µM/glucose 75 mM). We used a microscope at 10× magnification.

The pathology of the damage during diabetes retinopathy is the increase of oxidative stress. The DPPH analysis showed the high antioxidant ability of fucoxanthin (Figure 7A). We used a catalase assay kit and DCFDA to measure the change of oxidative stress. The results showed that the fucoxanthin, which has a high antioxidant ability according to a previous study [12], increased the catalase activity (Figure 7D,E) and decreased the ROS level (Figure 7B,C,E,F), which may alter the 4-HNE- and high glucose-induced oxidative stress. This demonstrates the antioxidant effect to recover the oxidative stress caused by 4-HNE- and high glucose-induced retinopathy.

## 4. Discussion

In the present study, we used two different models to simulate diabetes retinopathy. We discovered that fucoxanthin not only recovered the cell number of ARPE-19 but also alleviated the apoptosis, adhesion molecules, barrier completeness, and tight junction connection protein expression. We also discovered that the function of fucoxanthin in retinopathy is based on its antioxidant ability through the reduction of the ROS density.

Diabetes retinopathy may contribute 4–15% of the severe visual impairment in Western countries [21]. The high glucose condition of diabetes causes several complications [22], with diabetes retinopathy causing one of the most severe complications resulting in blindness in adults. To examine the potential compounds or supplements to relieve the symptoms is an important issue. ARPE-19 is widely used as the diabetes retinopathy in vitro model. To evaluate the hyperglycemia condition, we used high glucose treatments to examine diabetes retinopathy. High glucose at 70 mM significantly decreased the cell number of ARPE-19 through increased ROS [23], indicating that high glucose increased the ROS and reduced the antioxidant enzyme ability and that the persistent oxidative stress caused the retinal damage [24].

The increase of ROS can be related with the increase of inflammation [25,26] and apoptosis [27]. Enzymatic antioxidant defenses are controlled by the transcription factor Nrf2 [28]. The high glucose condition may decrease the Nrf2 protein expression, further eliminating the protective antioxidant related enzyme [28]. The persistent hyperglycemia may increase the lipid peroxidation effect [29], with the increased ROS causing ARPE-19 cell damage and cell death. Consistent with the present study, high glucose and lipid peroxidation demonstrated increased ROS, PARP, and Nrf2 and decreased catalase, showing the increase of apoptosis and the decrease of antioxidant enzymes resulting in the increase of ROS.

The blood–retinal barrier (BRB) provides protection of the retina. With high glucose or lipid peroxidation, there is strong evidence for the BRB breakdown and RPE dysfunction shown in diabetes retinopathy [30]. The decreased ZO-1 and occludin disrupt the tight junction in the retinal outer BRB. The hypoxia and high glucose condition showed a significant decrease in the expression of tight junction proteins with an increase of vascular endothelial growth factor (VEGF) and fibronectin to weaken the ARPE-19 cell monolayers [31]. The 4-HNE treatment showed a significant breakdown of the intestine barrier with decreased ZO-1 protein expression [32].

The breakdown of the tight junction increased the pro-inflammatory factors in the retina [33]. An ICAM-1 increase was related with retinal vascular hyper permeability, showing that the completeness of the retina is related with the tight junction and the vascular permeability. Intercellular adhesion molecule-1 (ICAM-1) is a member of the immunoglobulin supergene family with the ability of cellular adhesion molecules mediating the attachment of lymphocytes. Diabetes retinopathy patients in a meta-analysis research showed that ICAM-1 expression was related to the severity of diabetes retinopathy and generally exists in patients [34]. Our results are also consistent with the pathology of diabetes retinopathy, showing a decrease of tight junction-related protein expression and increased ICAM-1 in the high glucose- and 4-HNE-induced retinopathy.

The inhibition of the ROS is the major feature in diabetes retinopathy. Marine organisms are able to synthesize molecules with the antioxidant effect to protect themselves and avoid harm from ultraviolet radiation, oxidative stress, and environmental condition exposure [12]. With visible light-induced retinal damage, fucoxanthin, a special xanthophyll derived from edible brown seaweeds with a high antioxidant effect, modulates the glycometabolism and lipid metabolism giving a protection effect to the light-induced RPE cell damage with a decrease of the oxidative stress [11]. A previous study shows that fucoxanthin can alter the ROS-generating factors glucose-6-phosphate dehydrogenase (G6PDH) and NADPH oxidase 4 (NOX4) and alleviate the antioxidant enzyme related protein expression to suppress the ROS generation [35]. In the present study, fucoxanthin showed a high antioxidant ability in the reduction of ROS, and the increase in catalase decreased the apoptosis effect to protect from cell damage in high glucose and 4-HNE conditions.

In a Caco2 cell line treated with the lipopolysaccharides (LPS), fucoxanthin significantly recovered the LPS-induced tight junction loss by the recovery of the occludin and claudin [36]. The present study is, to our knowledge, the first research to discover fucoxanthin’s ability in the recovery of the blood–retinal barrier completeness and permeability with the recovery of ZO1 and occludin protein expression.

Carotenoids are natural antioxidants, which are found abundantly in fruits and vegetables. Examples include β-carotene, lutein, and zeaxanthin. A precursor of vitamin A with antioxidant ability could help maintain the oxidative stress, apoptosis, and inflammation to protect the health of the eyes, preventing eye diseases, such as cataracts, age-related macular degeneration, and diabetic retinopathy [37].

As a carotenoid, fucoxanthin shows a great ability to reduce oxidative stress and reduce retinopathy related pathology. In Caco-2 absorption, fucoxanthinol (FxOH) may rapidly hydrolyze from fucoxanthin in the gastrointestinal tract, and most metabolites are accumulated in adipose tissue [38]. However, some evidence also shows that the fucoxanthin absorption does not convert to any metabolites [39]. Whether the fucoxanthin can metabolize and form metabolites remains to be studied in further research. Furthermore, the limitation of our study is that the treatment we performed did not resemble the physiological function of fucoxanthin entering the cell. This indicates that there is still a need for an in vivo study to confirm the protection of fucoxanthin in diabetes retinopathy.

In this study, we demonstrated the potential of fucoxanthin for protection against diabetes retinopathy. We used two different kinds of diabetes retinopathy cell models and showed a consistent protective effect.

## 5. Conclusions

**Figure 8:**
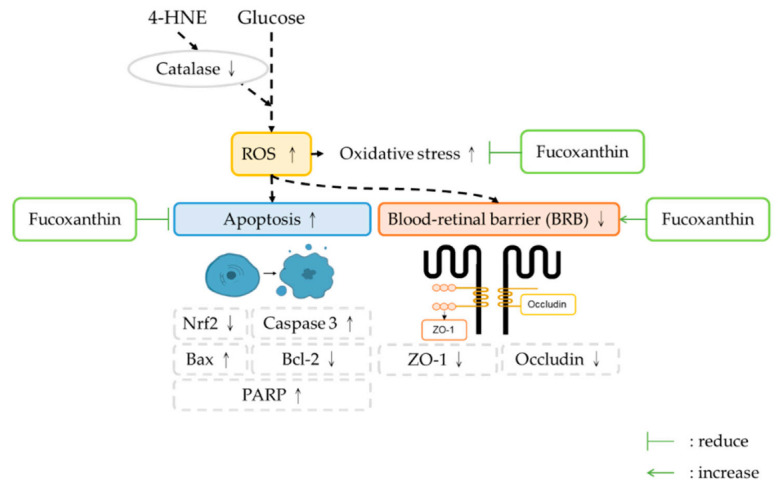
Schematic representation of the protective effect of fucoxanthin on the 4-HNE- and high glucose-induced ARPE19 cell damage. The reduction of the ROS and the completeness of the blood–retinal barrier provides structural integrity and indicate a protective ability of fucoxanthin in diabetes retinopathy.

The treatment of fucoxanthin was demonstrated to effectively protect against the effects of 4-HNE- and high glucose-induced diabetes retinopathy in human retinal epithelial cells (ARPE-19) through the antioxidant ability of fucoxanthin. (1) Fucoxanthin can decrease the ROS level by the increase of catalase activity. (2) Fucoxanthin can ameliorate the blood–retinal barrier completeness. (3) Fucoxanthin can reduce the apoptosis rate to decrease the damage caused by diabetes retinopathy. These data show that fucoxanthin has a potential effect on the protection of diabetes retinopathy (Figure 8).
